# Supplementary material for: The impact of vaccine-linked chemotherapy on liver health in a mouse model of chronic Trypanosoma cruzi infection
Source: PLoS Negl Trop Dis. 2023 Nov 21;17(11):e0011519. doi: 10.1371/journal.pntd.0011519 (PMC10697595; doi:10.1371/journal.pntd.0011519)
Supplement: Attachment — Submitted filename: 11-6-23 Response letter.pdf [file pntd.0011519.s001.pdf]

National School of
Tropical Medicine

One Baylor Plaza, BCM113
Houston, TX 77030-3411

713-798-1199
Fax 713-798-2299

November 6, 2023

To: Renata Rosito Tonelli, PhD, Academic Editor
Abhay Satoskar, Section Editor
PLOS Neglected Tropical Diseases

Re: Response to review of Manuscript PNTD-D-23-00863

Dear Dr. Tonelli and Dr. Satoskar,

On behalf of my co-authors I would like to thank you for the comprehensive and thorough review of Manuscript PNTD-D-23-00863 entitled "The impact of vaccine-linked chemotherapy on liver health in a mouse model of chronic Trypanosoma cruzi infection". We have carefully reviewed the comments from the reviewers and addressed each comment in this response letter and within the revised manuscript. We feel that this revised version is significantly improved and we hope it is now suitable for publication in PLOS NTD. Please do not hesitate to contact me if additional revisions are needed.

Sincerely,

Kathryn M. Jones, DVM, PhD
Associate Professor
National School of Tropical Medicine
Baylor College of Medicine
Houston, TX, USA
Reviewer's Responses to Questions

Key Review Criteria Required for Acceptance? As you describe the new analyses required for acceptance, please consider the following:

Methods
-Are the objectives of the study clearly articulated with a clear testable hypothesis stated?
-Is the study design appropriate to address the stated objectives?
-Is the population clearly described and appropriate for the hypothesis being tested?
-Is the sample size sufficient to ensure adequate power to address the hypothesis being tested?
-Were correct statistical analysis used to support conclusions?
-Are there concerns about ethical or regulatory requirements being met?

Reviewer #1: No issues

Reviewer #2: This study was designed to verified weather low doses of the anti-parasitic drug benznidazole (BNZ) combined with a prototype therapeutic vaccine is effective in controlling parasite infection without causing liver damage. BNZ is a drug known to cause liver damage if administered with a curative dose. In a previous study the authors showed that a combination of low dose plus a recombinant protein vaccine can effectively reduce T. cruzi induced cardiac damage. Here, the authors evaluated several markers of liver health after treatment with low dose BNZ plus the vaccine therapy. The authors showed that, as expected, treatment of infected mice with curative doses of BNZ reduces parasite burden but elevates serum levels of several toxicity markers. However, neither low doses of BNZ or low doses plus vaccine results in reduced parasite burden but low doses plus vaccine results in a slight better outcome in terms of liver toxicity compared to the effect of a curative dose of BNZ. This is a well conducted and useful study that may help optimize the treatment for Chagas disease. All the objectives of the study were clearly articulated with a testable hypothesis, and the study design appropriately addresses its objectives. Proper statistical analysis was used to support the conclusions and all ethical requirements regarding animal use have been met.

Response: We thank the reviewers for their critical review and their constructive feedback for improving this manuscript.

Results
-Does the analysis presented match the analysis plan?
-Are the results clearly and completely presented?
-Are the figures (Tables, Images) of sufficient quality for clarity?

Reviewer #1: See general comments

Reviewer #2:

The experiments were well designed, and the results of the analysis were clearly presented, with all figures having good quality. As indicated below, I have few criticisms regarding the lack of statistical significance analyses of part of the data, which would be important to strengthen some of the authors conclusions. Line 160, the authors used bioluminescent parasites but on line 180 they indicated that tissue parasite burden was determined only by quantitative real-time PCR. Please explain why luciferase expressing parasites were used. Also, regarding parasite burden, on Figure 3 it should be indicated how the parasite numbers were calculated (is it per mg of tissue?).

Response:

We elected to use a luciferase expressing clone of the *T. cruzi* H1 strain for these studies as we have demonstrated that chronic infection with this clone induces significant cardiac pathology in our female BALB/c mouse model, including changes in cardiac structure and function (Liu et al, 2023), similar to changes induced by chronic infection with the WT *T. cruzi* H1 strain used in prior studies (Jones et al, 2023). The Materials and Methods was revised to include this explanation. For this study, only quantitative PCR was used to measure tissue parasite levels to be comparable to prior published studies. Figure 3 has been revised to include the units of measure for both the parasites in panel 3A and the inflammatory cells in panel 3B. (See lines 145-149).

Reviewer #2:

Fig 2, line 238, the authors claimed that "these data confirm hepatomegaly is evident in our model and that only curative BNZ ameliorates this finding." However, the difference in the liver weight/body weight is minimal. Therefore, the sentence should be changed to "these data confirm hepatomegaly is evident in our model and that

curative BNZ slightly ameliorates this finding". None of the other treatments had an impact in liver health based on the liver weight/body weight ratio. Why the liver weight/body weight ratio was not measured in animals treated with low plus vaccine and curative BNZ doses at 90 dpi, when this ration was much higher compared to the ration at 142 dpi?

Response:

Thank you for your observation. We agree that the difference in liver weight/body weight ratio between infected untreated mice at 142dpi (Figure 2C purple symbols) and infected mice treated with a curative dose of BNZ at 142dpi (Figure 2C dark brown symbols) is small, however statistical analysis revealed that this difference is statistically significant. Additionally, curative BNZ does not restore the liver weight/body weight ratio to the same level as age matched naïve mice. We have modified the results to state that "These data confirm hepatomegaly is evident in our mouse model and that curative BNZ reduces infection induced hepatomegaly by 142dpi, but does not restore liver weight/body weight ratio to the same level as age matched naïve mice". (See lines 244-247)

Regarding evaluating the impact of low BNZ + vaccine compared to curative BNZ at earlier time points, we could not measure liver weight/body weight ratios at 90 dpi due to the study timeline. We used a sequential treatment strategy where beginning at approximately 70 dpi mice were given once daily treatment with BNZ for 18 consecutive days, then vaccine was administered twice, two weeks apart. Therefore, for the low-BNZ + vaccine treatment, the boost vaccination was not administered until approximately 100 days post infection. Further, we have found that the impact of low-BNZ + vaccine treatment has the most beneficial effects on cardiac structure and function between approximately 128dpi to 156 dpi (Jones et al, 2023 DOI: 10.3389/fcimb.2023.1106315). Therefore, we elected to evaluate the impact of low BNZ +vaccine and curative BNZ at 142dpi for this study.

Reviewer #2:

Fig 3, lane 254: are there statistically significance differences in inflammatory infiltrate between infected untreated at 90 dpi and 120 dpi? Or between naïve and infected untreated at 142 dpi? Please indicate in the text. Again, like the liver weight/body weight ratio, the main increase in inflammatory infiltrate is observed at 90 dpi. Why was the effect of curative BNZ only measured 142 dpi when just a small decrease compared to infected untreated mice was observed? Also, the authors claims that curative BNZ significantly decreased inflammatory infiltrate compared to infected untreated mice, however, it was not clear whether the inflammatory infiltrate in the liver of untreated infected mice was increased compared to naïve mice.

Response:

Thank you for your observation. We modified Figure 3B and the results to clarify that at 142dpi, infection induced significantly increased inflammatory infiltrate compared to naïve age matched controls, and this increase was significantly reduced by curative BNZ treatment. Additionally, at 120dpi, inflammatory infiltrate was significantly reduced by infection compared to 90dpi (Figure 3B green and red symbols respectively) however there was no statistically significant difference in inflammatory infiltrate when comparing 90dpi to 142dpi. (See figure 3 and lines 258-265)

We acknowledge that in our model it appears that the greatest increase in levels of inflammatory cell infiltration into the liver is at 90dpi. The purpose of the current study was to compare the effects of low BNZ+ vaccine and curative BNZ on liver health, which due to the required timeline for low BNZ+ vaccine treatment precluded evaluating impacts at 90 dpi because the low BNZ + vaccine treatment was not completed at that time. In future studies we will evaluate the impact of curative BNZ treatment on liver inflammation and liver health at multiple timepoints, including immediately after treatment, to determine the kinetics of the responses.

Reviewer #2:

Fig 5, lane 273: are there statistically significance differences in levels of ALT and AST between naïve and infected untreated mice at any dpi? If not, the observations that both curative BNZ and low BNZ + vaccine induced significant elevations to ALT by 142dpi, compared to naïve animals may suggests that the liver damage occurs in response to the treatment and not to the infection. Increased tissue damage was detected when infected and curative BNZ treated mice was compared to mice infected at the same dpi. Based solely on this, the authors could not claim that the "vaccine-linked chemotherapy strategy causes less liver and tissue damage compared to curative BNZ alone"

Response:
We did not observe any statistically significant differences in ALT or AST levels when comparing naïve mice to infected mice at any timepoint. However, we did observe that mice that received either low BNZ+ vaccine or curative BNZ did have significantly elevated ALT and AST levels compared to naïve mice. We agree that it is likely the combined effect of curative BNZ and infection that caused significant elevation in ALT and AST when compared to age matched naïve mice. Our finding in this paper that infected mice treated with curative BNZ have higher levels of these liver damage enzymes is consistent with the published report of Novaes et al, 2015, where they demonstrated that **the combined effect of infection and BNZ treatment resulted in greater elevations in liver enzymes and direct liver damage compared to either infection or BNZ treatment** of naïve mice alone. DOI: 10.1007/s00436-015-4488-x. Importantly, as shown in figure 5 D, only curative BZN treatment caused elevation in another important damage marker, LDH, compared to age matched naïve mice. Low BNZ + Vaccine did not cause a significant elevation in LDH. It is because low BNZ + vaccine only caused elevations in ALT and AST compared to naïve mice, where curative BNZ caused elevations in ALT, AST and LDH that we concluded that the data suggests low BNZ + vaccine is less damaging to the liver when compared to curative BNZ. Our interpretation of this data is included in the discussion section. (See Lines 355-361)
We acknowledge that in future studies it will be important to include uninfected mice treated with low BNZ, curative BNZ, vaccine and low BNZ + vaccine to determine the impact of these treatments alone on liver damage, however that was outside the scope of the present study.

Reviewer #2:

Fig 6, line 289: In contrast to the results obtained with enzyme assays, it looks like the authors were able to observe signs of increased liver damage in infected untreated animals at 120 dpi compared to 90 dpi and when 142 dpi was compared to 90 dpi using the expression of a marker of oxidative damage (BTG2) and a regulator of inflammation (PPARα). Again, are these differences statistically significant? If so, it should be mentioned. It should be also mentioned that although low BNZ treatment can reduce oxidative damage and regulation of inflammation, low BNZ + vaccine does not contribute for a reduction of these parameters the same way that this treatment does not cause a reduction in inflammatory infiltrate.

Response:

Thank you for your observation. We have revised figure 6 to include the statistical comparisons of both BTG2 and PPARα expression in infected mice at different timepoints, and these results are clarified in the text. We also modified our statement at the end of the results to clarify that while these data show that low BNZ may improve oxidative damage and regulation of inflammation, the combination of low BNZ + vaccine does not result in similar improvement. (See Figure 6 and lines 302-312)

Conclusions
-Are the conclusions supported by the data presented?
-Are the limitations of analysis clearly described?
-Do the authors discuss how these data can be helpful to advance our understanding of the topic under study?
-Is public health relevance addressed?

Reviewer #1: See general comments

Reviewer #2:

Most of the authors conclusions are supported by the data presented, but, for the sake of clarity, in the abstract, the conclusion sentence should be modified to "These data confirm toxicity associated with curative doses of BNZ and suggest that, although the dose sparing low BNZ plus vaccine treatment does not reduce parasite burden, it better preserves liver health."

Response:

Thank you for your comment. We have modified the abstract as suggested.

Editorial and Data Presentation Modifications?

Use this section for editorial suggestions as well as relatively minor modifications of existing data that would enhance clarity. If the only modifications needed are minor and/or editorial, you may wish to recommend "Minor Revision" or "Accept".

Reviewer #1: See general comments

Reviewer #2: no comments

Summary and General Comments
Use this section to provide overall comments, discuss strengths/weaknesses of the study, novelty, significance, general execution and scholarship. You may also include additional comments for the author, including concerns about dual publication, research ethics, or publication ethics. If requesting major revision, please articulate the new experiments that are needed.

Reviewer #1:

This paper focuses on liver toxicity issues during Chagas disease and during therapy using a mouse model. Parasite infected animals are compared with infected animals treated with either high-dose benznidazole (BNZ) therapy and those treated with low-dose BNZ+vaccine, a previously described subunit vaccine. This latter combination has been shown to result in decrease cardiac disease while have the advantage of reduced exposure to the toxicities of high dose BNZ monotherapy. Overall, the study found that high dose BNZ reduced liver parasite burden and inflammation but leads to liver toxicity as measured liver and tissues enzyme elevations. Low-dose BNZ+vaccine dose not reduce parasite burdens or tissue inflammation but results in similar toxicity to HD-BNZ whereas LD-BNZ alone or vaccine alone does not.

Overall the experiments are performed well and the data justifies the conclusions.

1) The major issue is that while the LD BNZ-vaccine combo appears to not have significant liver toxicity, it does not have any impact on parasites in the liver. And while the use of this treatment may indeed be important on cardiac disease it does not treat Chagas liver disease. Whether it reduces or ameliorates parasitization of other tissues is unknown. By extension, how does one treat Chagas liver disease without high dose BNZ? If using LD-BNZ+vaccine for cardiac disease, one would still need another agent to treat disease in the liver or other organs.

Response:

We thank you for your observation. We agree that the data presented here does not show that the low BNZ + vaccine treatment eliminates liver parasites or significantly reduce inflammation in the liver. However, since we have previously demonstrated that low BNZ + vaccine improves cardiac structure, function and inflammation, and more recently we showed that low BNZ + vaccine better restores cardiac metabolism when compared to curative BNZ treatment Jones et al, 2023 DOI: 10.3389/fcimb.2023.1106315; Liu et al, 2023 DOI: 10.1038/s41467-023-42247-w; the purpose of this study was to evaluate the impact of this treatment on liver health as it is well known that curative doses of BNZ cause significant toxicity. From the data we present, we conclude that low BNZ + vaccine is less damaging to the liver compared to curative BNZ, so with the benefit of this treatment on cardiac health it is a promising strategy to pursue for clinical development. However, additional studies are necessary to determine the impact of this treatment on other organs, and to optimize a multimodal treatment strategy that would better treat liver disease specifically in Chagas disease. We modified the discussion (lines 446-450) to discuss the need for additional studies to develop strategies to further improve liver health.

Reviewer #1:

2) Both HD-BNZ and LD-BNZ+vaccine treatment induces ALT, AST release from liver of infected mice when compared to naive controls (Fig. 5 A and B). There are no controls of naïve mice treated with either drug alone. In the case of HD-BNZ this could be interpreted as turnover of parasites in the liver affecting hepatocyte toxicity. In the case of LD-BNZ+vaccine there is no demonstrable effect on parasite turnover yet there is still liver toxicity. Why do the authors conclude that this treatment doesn't produce liver toxicity (lines 279-280 and in line 50), but later state that this isn't the case (356-359)? LD-BNZ alone or vaccine alone did not result in AST/ALT elevations the combo did leading one to conclude that these synergize to induce toxicity during dual therapy. Perhaps the authors should elaborate on how this may happen.

Response:

Thank you for your observation. We acknowledge that to definitively define the relative contributions of low BNZ, vaccine, and curative BNZ on liver health we would need to include groups of uninfected mice given each treatment to compare to infected mice that are untreated or given the treatments. However, that was outside the scope of the present study.

Regarding the impact of low BNZ + vaccine on liver health, we do not conclude that there is no toxicity associated with the combination treatment, only that the treatment causes less toxicity compared to curative BNZ and better preserves liver health. In the discussion we state "However, the group treated with low BNZ + vaccine sequentially had elevated serum levels of ALT and AST at 142dpi, similar to curative BNZ. **This suggests that despite the dose sparing effect of this strategy, the combination of treatments does still result in elevation of tissue damage enzymes"** (see lines 371-375)

We propose that the elevations in ALT and AST due to low BNZ + vaccine treatment could be due to the combined effects of reactive metabolites resulting from BNZ treatment and activation of inflammatory pathways by E6020, the TLR4 agonist adjuvants used in the vaccine component (See lines 373-375)

Reviewer #1:

3) Regarding the statistics, it is hard to understand why some groups are not statistically different than others just based on the visual representation. For example, Fig 5A, LD-BNZ alone and vaccine alone look even more statistically different to either LD-BNZ+vaccine or HD-BNZ than does naïve mice and LD-BNZ+vaccine or HD-BNZ. Why are they not?

Response:

Thank you for your comment. Our ultimate goal was to compare the impact of treatments to infected untreated mice at each timepoint. We clarified this in the materials and methods (See lines 223-225) Therefore we did not directly compare LD-BNZ alone to LD BNZ+ vaccine because those samples were taken at different times after infection (as indicated in the figures) and would not effectively capture the combined effects of duration of infection and treatment effects at each timepoint. We acknowledge in the discussion that limited timepoints were included in this study to evaluate immediate effects of treatment, and additional timepoints will need to evaluated in future studies (See lines 431-433). However, in the revised manuscript we did compare infected untreated mice at different timepoints to characterize the changes induced by infection over time (See figures 3 and 6).

Reviewer #1:

4) There are some minor grammatical issues: line 357, 375, different fonts between lines 443-445.

Response:

Thank you for your comment. We have reviewed the manuscript and edited it to correct grammatical errors and formatting.

Reviewer #2: please see comments above.
